# Surround-Masking Affects Visual Estimation Ability

**DOI:** 10.3389/fnint.2017.00007

**Published:** 2017-03-16

**Authors:** Nicola R. Jastrzebski, Laila E. Hugrass, Sheila G. Crewther, David P. Crewther

**Affiliations:** ^1^Centre for Human Psychopharmacology, Faculty of Health, Arts and Design, Swinburne University of TechnologyHawthorn, VIC, Australia; ^2^School of Psychological Sciences, La Trobe UniversityBundoora, VIC, Australia

**Keywords:** visual estimation, contrast saturation, surround inhibition, sensory filtering, number acuity

## Abstract

Visual estimation of numerosity involves the discrimination of magnitude between two distributions or perceptual sets that vary in number of elements. How performance on such estimation depends on peripheral sensory stimulation is unclear, even in typically developing adults. Here, we varied the central and surround contrast of stimuli that comprised a visual estimation task in order to determine whether mechanisms involved with the removal of unessential visual input functionally contributes toward number acuity. The visual estimation judgments of typically developed adults were significantly impaired for high but not low contrast surround stimulus conditions. The center and surround contrasts of the stimuli also differentially affected the accuracy of numerosity estimation depending on whether fewer or more dots were presented. Remarkably, observers demonstrated the highest mean percentage accuracy across stimulus conditions in the discrimination of more elements when the surround contrast was low and the background luminance of the central region containing the elements was dark (black center). Conversely, accuracy was severely impaired during the discrimination of fewer elements when the surround contrast was high and the background luminance of the central region was mid level (gray center). These findings suggest that estimation ability is functionally related to the quality of low-order filtration of unessential visual information. These surround masking results may help understanding of the poor visual estimation ability commonly observed in developmental dyscalculia.

## Introduction

The effect of peripheral visual stimulation, varying in contrast and or luminance, on the ability to make numerosity estimation judgments of centrally presented elements, has seldom been investigated in typically developing adults. The process of visual estimation involves the discrimination of magnitude between two distributions or perceptual sets that vary in number of elements. Peripheral visual stimulation at high sensory load deleteriously affects such discrimination. Examples of peripheral involvement include discrimination of texture regions (Xing and Heeger, 2000, 2001; Dakin et al., 2005), tilt of line bars or Gabor elements (Polat and Norcia, 1996; Van Der Smagt et al., 2005), and awareness of motion direction (Tadin et al., 2003, 2006; Tadin and Lappin, 2005). The perceptual inefficiencies induced by surround masking have been related to low-order inhibitory mechanisms at the neural level as reported for surround suppression in lateral geniculate nucleus (LGN) or intra-cortical inhibition in V1 and extra-striate regions (Carandini et al., 2002; Shapley et al., 2003; Carandini, 2004). One of the earliest (and most influential) investigations into the effects of surround-masking (Chubb et al., 1989) revealed that the perceptual contrast of a central texture region became much lower when enveloped by a high contrast surround.

The Chubb et al. (1989) investigation initiated inquiry into the relationship between induced perceptual inefficiency and inhibitory gain control during the attenuation of redundant visual information. For example, Xing and Heeger (2000) replicated the findings of the Chubb et al. (1989) surround-masking experiments, and found that a low contrast annular grating had a facilitative effect on the apparent contrast of the centrally embedded texture patch. In the presence of a low contrast annular grating, the perceived contrast of the centrally embedded texture was markedly higher than its contrast matched reference patch without a surround. In further investigation of this effect, Xing and Heeger (2001) observed that surround-masking psychophysical performance also was influenced by the width and orientation of the surround annulus—a high contrast surround (80%) with a diameter of 12° produced the greatest level of suppression and reduced psychophysical performance for all observers. The suppressive effects were reversed, however, when the surround annulus diameter was narrowed to 7°—even though the annulus contrast was high (80%).

Xing and Heeger (2001) noted that the psychophysical properties of receptive field (RF) excitation (facilitation) and RF inhibition (suppression) depended upon the contrast of central and surround stimuli and the diameter of the surround annulus. Excitatory RF processes were postulated to be dominant under low contrast/narrow surround diameter stimulus conditions—occurring mainly within the foveal field of vision. Inhibitory RF processes, on the other hand, were argued to be dominant under high contrast/extended surround stimulus configurations—occurring chiefly in the peripheral field of vision. In line with these findings, Tadin et al. (2003, 2006) observed that the inspection time for motion direction discrimination of drifting Gabor patches showed a strong interaction between stimulus contrast and stimulus size. While for small patches, duration thresholds were smallest for high contrast gratings, the opposite was true for large patches—optimal motion discrimination occurred under low contrast conditions.

The functional role of RF inhibition in V1 includes the attenuation of afferent input with high contrast gain (Webb et al., 2005); the gating of statistically redundant afferent information—sensory filtering (Schwartz and Simoncelli, 2001) and feature segmentation modulated by orientation selectivity (Shapley et al., 2003). Multi-unit recordings of anesthetized cats (Freeman et al., 2002) suggest RF inhibition in V1 is likely to be relayed from LGN, through mechanisms involving thalamo-cortical synaptic depression (Carandini et al., 2002).

From the computational modeling, primate neurophysiology and psychophysical literature on visual suppressive phenomena, it has been suggested that the RF suppression of cells in LGN and V1 through surround-masking is likely to suppress sensory gain control resources, thereby reducing the signal to noise ratio (SNR) of sensory/afferent information. This, in turn, would generate perceptual ambiguity and representational noisiness (Dosher and Lu, 1998, 2000; Lu and Dosher, 1998, 1999, 2008; Schwartz and Simoncelli, 2001; Carandini et al., 2002; Freeman et al., 2002; Dakin et al., 2005; Webb et al., 2005). Preliminary electrophysiological evidence from non-linear visual evoked potential (VEP) recordings suggests that those with sub-optimal arithmetical ability of developmental origin show disinhibited sensory gain control mechanisms as well as impoverished change detection performance under high contrast conditions (Jastrzebski et al., 2015).

An earlier psychophysical study demonstrated that children with low mathematical skills show higher motion coherence discrimination thresholds than age-matched controls (Sigmundsson et al., 2010), suggesting that developmental dyscalculia (DD)—poor arithmetical ability despite normal intelligence, may be associated with a visual perceptual disorder in contrast gain control or external noise exclusion (Carandini, 2004; Sperling et al., 2005). Curiously, many developmental disorders that are characterized by poorer academic performance such as autism spectrum disorder (ASD), attention deficit hyperactivity disorder, Williams syndrome (WS), and developmental dyslexia share a common perceptual deficit in motion coherence discrimination—particularly for global motion coherence stimulus configurations (Cornelissen et al., 1998; Stein et al., 2000; Laycock et al., 2007; Braddick and Atkinson, 2011), with a possible common feature of atypical development of the dorsal visual stream.

The acquisition of visual number estimation skills for individuals with WS—a neurodevelopmental disorder strongly associated with DD—has been shown to be more variable and delayed over the course of developmental time compared to neurotypical age-matched controls (Ansari et al., 2007). Over the course of development for the typically developing group, there was a graded increase in the mean proportion of correct responses (PCR) and concomitant decrease in the coefficient of variation (COV), while the WS adult group only showed a marginal performance increase and very little decrease in the COV. The rationale for the comparison of WS and typically developed (TD) visual number estimation skills was to examine which developmental and low-level factors contributed to poor estimation other than mental age or IQ across developmental time. That is, the adult and child participants with WS had mental ages much lower than the comparison TD adult and child participants. Here, poorer visual estimation performance across developmental time of adult WS participants was attributed to low-level factors that caused disturbances in perceptual development. While the investigators did not explicitly specify the functional characteristics of “low-level perceptual disturbance” in WS, an inferential link can nonetheless be established with atypical development of the dorsal visual pathway, given that the WS phenotype is characterized by such “low-level” perceptual disturbances (Braddick and Atkinson, 2011).

Similar to the individuals with WS (Ansari et al., 2007), Piazza et al. (2010) also noted an absence of improvement of visual estimation ability (number acuity) from early to late childhood of those diagnosed with DD. By contrast, the age-matched control group demonstrated decreased number acuity thresholds (Weber fractions) across developmental time. The Weber fractions of 10-year-old DD observers were not unlike those of the 5-year-old typically developing children. A longitudinal study (Halberda et al., 2008) noted that early childhood proficiency with visual estimation ability was the best predictor of later symbolic math achievement during early adolescence (14 years)—even when other factors contributing to math achievement such as general intelligence were controlled for.

Thus, there is evidence to suggest that visual estimation ability in early childhood predicts later mathematical achievement during the course of cognitive development (Halberda et al., 2008). But what are the visuo-perceptual and developmental factors that predict this sense of number acuity? According to Gallistel and Gelman (2000), the perceptual noisiness during visual estimation follows Weber’s law, where the discriminability between two numerosities become more impoverished as the magnitude difference (ratio) between them decreases, and the number of elements within the distribution increases. In other words, more numerous set sizes with smaller differences between them will result in overlap of the signal distributions that represent the numeric perceptual sets—the scalar variability.

Could the scalar variability (noisiness) of visual estimation be influenced by the functional quality of inhibitory gain control mechanisms discussed earlier? We sought to answer this question directly via investigation of the effects of high contrast surround-masking on visual estimation performance in neurotypical observers. If high contrast surround-masking causes impairment of visual estimation accuracy of neurotypical observers, it shows cause to infer that the poor number acuity previously observed in DD (cf. Piazza et al., 2010) may stem from developmentally anomalous inhibitory gain control mechanisms that play a major role in the elimination of redundant visual information, i.e., perceptual noise exclusion (Schwartz and Simoncelli, 2001; Carandini et al., 2002; Dakin et al., 2005; Lu and Dosher, 2008).

## Materials and Methods

### Participants

Eighteen young adults with normal/corrected to normal vision [mean age ± standard deviation (SD) = 23.8 ± 6.06 years, 13 females] participated in this experiment. This sample mostly comprised undergraduate psychology students who were awarded course credit for their participation, and post-graduate students who participated voluntarily without compensation. The study was carried out in accordance with the Helsinki declaration and approved by the Swinburne University of Technology ethics committee. Written informed consent was obtained from all subjects. Upon inspection of the raw data, it was apparent that there were two individuals with psychophysical responses that were ±2 SDs from the mean, which were markedly deviant from the remaining 16 participants and hence were excluded from the analysis.

### Stimuli

The stimuli were generated using VPixx software (version 2.79—www.vpixx.com), presented on a 1680 × 1050 pixel Mac Pro cinema display with a frame rate of 60 Hz, and viewed at a distance of 50 cm. The three main parametric variations of these experiments were background luminance of the central stimulus region (CSR; gray or black); visual estimation of number without an annular surround (gray background luminance or black background); and surround dot contrast (low 25% and high 95%). The CSR was a 6.5° × 6.5° aperture containing white dots (luminance 168.4 cd/m^2^, size 10 × 10 pixel), drifting randomly inside the CSR at 2.14°/s. Interleaved with the frames of the CSR and dots, was additive random dynamic binary noise (RDBN) of 0.2° granularity, giving the appearance of transparent noise. For the zero luminance CSR, the additive RDBN was at 90% contrast, and at 20% for uniform (gray). The mean luminances for black, gray, and white colors were 0.30, 40.1, and 168.3 cd/m^2^, respectively.

When observers made their visual estimation judgments, the CSR was enveloped by either a high or low contrast annulus having an outer radius of 17.5° and inner radius of 3.4° and filled with RDBN—also of 0.2° granularity. There was no overlap between the CSR and surround. Because additive RDBN was interleaved with the dots, the mean luminance for CSR and surround were different. Therefore, separate mean luminance measurements were taken for the CSR and surround. This revealed that the mean luminance for the uniform/gray CSR was 44.0 cd/m^2^, and 24.3 cd/m^2^ for the black CSR. The mean luminance of the high contrast surround was 89.69 cd/m^2^, and 40.39 cd/m^2^ for the low contrast surround. The range of dot numerosities was 8–104, where the minimum value of dots that could be displayed within the initial (reference) CSR was 8 and the maximum for estimating more dots was 104.

**FIGURE 1:**
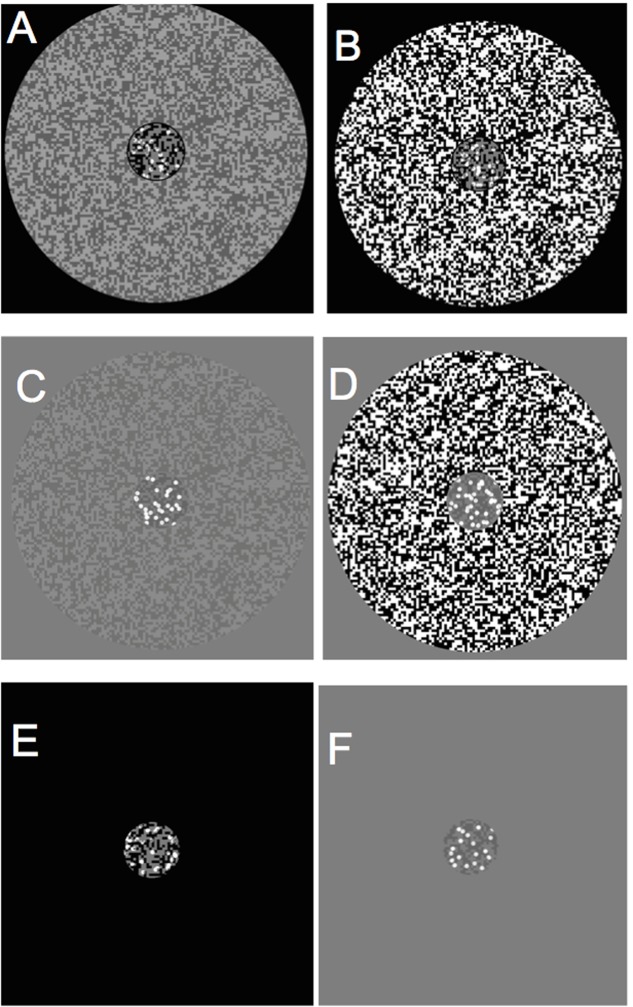
**Center and surround contrast stimulus configurations. (A)** Black central stimulus region(CSR) /low contrast surround, **(B)** black CSR/high contrast surround, **(C)** gray CSR/low contrast surround, **(D)** CSR/high contrast surround, **(E)** black CSR/zero luminance background, **(F)** gray CSR/uniform luminance background.

As seen from **Figure 1**, there were six center/surround configurations making up the experiment. For each experimental run, there were 50 trials per condition (i.e., 50 trials with fewer dots than the reference, and 50 trials with more dots, respectively). Hence, the entire experiment comprised a total of six separate runs using the method of constant stimuli, which was parametrically varied by CSR background luminance (uniform/zero); visual estimation with no surround, where the background luminance matched the CSR; and surround contrast (high/low). Participants took brief rests between experimental runs in order to minimize fatigue.

**FIGURE 2:**
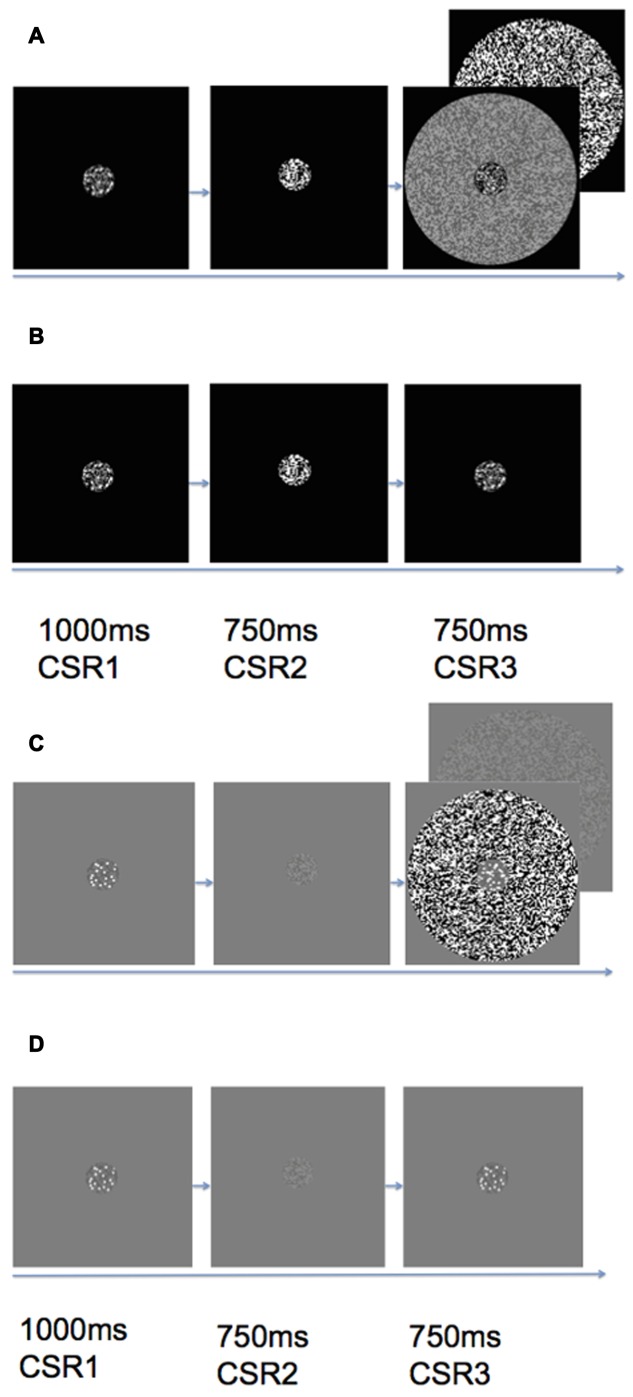
**Trial sequence of (A)** black center high/low contrast surround, **(B)** black center zero luminance background (no surround), **(C)** gray center high/low contrast surround, **(D)** gray center uniform luminance background (no surround). Each trial would begin with a CSR that contained a reference set of dots (CSR1) that appeared on the screen for 1 s. The CSR1 was then replaced by random dynamic binary noise (CSR2) that served as an inter-stimulus interval of 750 ms. The final stimulus presentation within a trial was CSR3, which contained a second set of dots either enveloped by a high or low contrast surround (see **A** and **C**), or no surround (see **B** and **D**). Observers made speeded responses as to whether there were more or less dots in CSR3 as compared to CSR1.

As can be seen in **Figure 2**, the stimulus sequence within an experimental trial contained three CSR stimuli at different times. The first CSR to appear within the trial sequence contained the reference set of dots, which remained on the screen for 1000 ms (CSR1). The second CSR (CSR2) was replaced with RDBN that was matched to the contrast of the annular-surround that served as an inter-stimulus interval of 750 ms. The final CSR to appear in a trial—like CSR1, also contained a set of dots, however, this time, the CSR was embedded in a high or low contrast annulus (CSR3). The difference ratio of dots between CSR1 and CSR3 was held constant at 1:0.5 throughout all experimental conditions.

### Procedure

Numerosity estimation is affected by many variables. Here, we investigated what effect contrast gain saturation had on the accuracy of numerosity estimation judgments via a series of 2 × 2 factorial designs that varied in central contrast, surround contrast, no surround, and numerosity. The objective of these experiments was to indicate by two alternate forced choice (2AFC) method whether CSR3 contained a fewer or more dots than CSR1. Observers were instructed to fixate upon the CSRs, and to indicate their responses after the onset of CSR3. Participants were instructed to respond as quickly and as accurately as possible. The experiments were performed in a darkened room without a chinrest, and each experimental run was counterbalanced across subjects in order to control for the effects of fatigue.

### Data Analysis

For simplicity, only the mean PCR for visual estimation of more and less dots across experimental conditions were examined. The reaction time (RT) data was not included in this analysis given that there were no significant effects found for numerosity, center or surround contrast. This absence in significant RT data was likely attributed to the brief exposure time of the CSR3 stimuli (750 ms), where observers had to rapidly make estimation judgments before the trial lapsed.

Differences in PCR were compared through a two-way, within-subjects analysis of variance (ANOVA), with focus on the effect that surround contrast (high/low/none) had upon the PCR for visual estimation of more and less dots. Subsequent ANOVAs explored the combination of other factors thought to affect visual estimation ability, such as center contrast (gray center/black center), as a more detailed exploration into how various combinations in center-surround contrast influence the PCR, and to note any variables other than surround contrast which may have confounded the effects of reduced PCRs observed in this study. *Post hoc* comparisons of significant simple effects were performed via two-tailed paired *t*-tests. There were no Bonferroni corrections applied to the paired *t*-tests because multiple comparisons were not performed.

## Results

### Effect of Surround Contrast and Set Size

**FIGURE 3:**
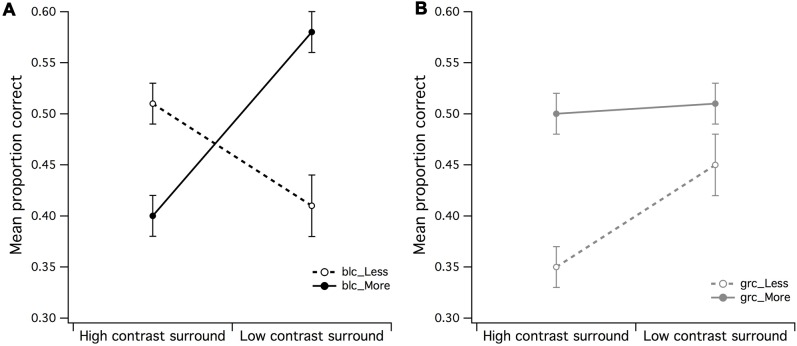
**Main effects of surround contrast and set of dots. (A)** There was a highly significant surround contrast by dot set/number interaction for black center and 1:0.5 difference ratio, where under high contrast surround conditions, observers had much more difficulty in accurate discrimination of more dots in CSR3, **(B)** the gray center and 1:0.5 difference ratio made it significantly more difficult for observers to accurately discriminate less dots in CSR3 under high contrast surround conditions. The vertical bars at the end of each line graph represent the standard error of the mean (SE) for **(A)** mean proportion of correct responses (PCR) for black center (less/more dots), and **(B)** mean PCR for gray center (less/more dots).

There were eight 2 by 2 within-subject ANOVAs performed, with the mean and SD of the PCR across each condition shown in **Table 1**, with *F* ratio and significance values summarized in **Table 2**. A 2 (high/mid surround contrast) by 2 (fewer/more dots) within-subjects ANOVA of black center stimuli revealed a significant main effect for surround contrast [*F*(1,15) = 5.75, *p* = 0.03, partial η^2^ = 0.28], and no significant effect for set size of dots under black center conditions (blc) (see **Figure 3A**). The surround contrast by dot set interaction was nonetheless highly significant [*F*(1,15) = 37.33, *p* < 0.001, partial η^2^ = 0.71]. *Post hoc* comparisons (paired *t*-tests) for this interaction revealed that the mean PCR for estimating less dots in the presence of the low contrast surround, was significantly lower than estimating more dots under the same surround contrast conditions [*t*(15) = 2.38, *p* = 0.002]. Under the high contrast surround condition the mean PCR for estimation of more dots was significantly lower than estimation of less dots [*t*(15) = 3.68, *p* = 0.031].

**Table 1:** Mean and standard deviation (SD) for proportion of correct responses.

	Center			
	Gray center (grc)		Black center (blc)	
Surround	Mean	*SD*	Mean	*SD*
**Low contrast**				
Less dots	0.45	0.14	0.41	0.13
More dots	0.51	0.13	0.58	0.09
**High contrast**				
Less dots	0.35	0.12	0.51	0.10
More dots	0.50	0.16	0.40	0.10
**No surround**				
Less dots	0.39	0.11	0.44	0.13
More dots	0.54	0.14	0.50	0.11

*N = 16*.

**Table 2:** Factorial design summary of 2 by 2 within-subjects ANOVAs.

Effects	ANOVA factors	*F*	*p*-value
1. Effect of surround contrast and set size	Black center:		
	Surround contrast (high/mid) by	5.75	0.03
	Number (less/more dots)	0.447	0.51
	Interaction	37.33	<0.001
	Gray center:		
	Surround contrast (high/mid) by	4.95	0.04
	Number (less/more dots)	3.74	0.07
	Interaction	1.45	0.25
2. Effect of surround contrast and center contrast	Less dots:		
	Surround contrast (high/mid) by	0.10	0.92
	Center contrast (high/mid)	5.83	0.03
	Interaction	21.76	<0.001
	More dots:		
	Surround contrast (high/mid) by	11.81	0.004
	Center contrast (high/mid)	0.75	0.40
	Interaction	10.69	0.005
3. Effect of background contrast and dot set size	Mean proportion of correct responses:		
	Background contrast (gray/black) by	0.001	0.98
	Number (less/more dots)	6.21	0.02
	Interaction	1.83	0.19
4. Effect of surround/black background and dot set Size	Black center:		
	High contrast surround/no surround (blc) by	2.02	0.17
	Less dots/more dots	0.361	0.55
	Interaction	8.35	0.01
5. Effects of surround/gray background and dot set size	Gray center:		
	Low contrast surround/no surround (grc) by	2.63	0.12
	Less dots/more dots	7.07	0.02
	Interaction	3.46	0.08
	Gray center:		
	High contrast surround/no surround (grc) by	4.16	0.06
	Less dots/more dots	9.25	0.008
	Interaction	0.02	0.886

A 2 (high/mid surround contrast) by 2 (fewer/more dots) within-subjects ANOVA of gray center stimuli revealed marginally significant main effect for surround contrast with gray center (grc) during visual estimation of dots (**Figure 3B**), where under high contrast surround conditions, the PCR for more and less dots were at or below chance level [*F*(1,15) = 4.94, *p* = 0.042, partial η^2^ = 0.25]. There was no significant effect for number, or significant surround contrast by dot set interaction (*F* < 1). Considered as a whole, these results suggest that for the black center background condition, the high contrast surround had the strongest impact upon the ability to discriminate whether there were more or less dots in CSR3 compared to CSR1.

### Effect of Surround Contrast and Center Contrast

**FIGURE 4:**
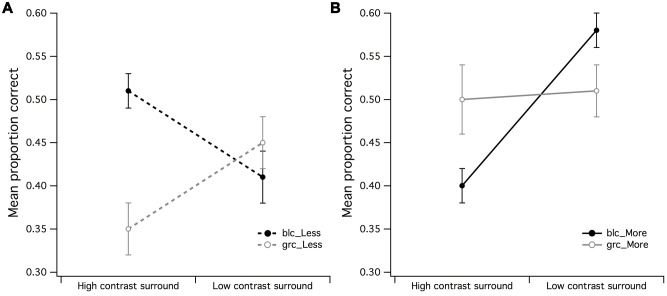
**Main effects of surround contrast by center contrast. (A)** The main effect for surround contrast during estimation of fewer dots with a 1:0.5 difference ratio was not significant, where it was much more difficult for observers to accurately estimate fewer dots under high contrast surround conditions with gray center than what it was for the black center, **(B)** the significant center contrast by surround contrast for estimation of more dots with 1:0.5 difference ratio suggested that the black center/high contrast surround configuration may have made observers perceive fewer dots in CSR3 than its veridical representation. The vertical bars at the end of each line graph represent the SE for **(A)** mean PCR for fewer dots (black and gray center), and **(B)** mean PCR for more dots (black and gray center).

A series of 2 (surround contrast) by 2 (center contrast) within-subjects ANOVAs were performed to examine whether the effects of visual estimation ability observed in **Figures 3A,B** were influenced by the contrast of the CSR. A surround contrast (high/low) by center contrast (grc/blc background) ANOVA for the PCR of less dots revealed a significant main effect for center contrast [*F*(1,15) = 5.83, *p* = 0.029, partial η^2^ = 0.28] but not surround contrast (**Figure 4A**). In addition, the surround contrast by center contrast interaction observed in **Figure 4A** was highly significant [*F*(1,15) = 21.76, *p* < 0.001, partial η^2^ = 0.59], implying that the mean PCR for estimation of “less” under the gray center and high contrast surround condition, was substantially lower than the blc and high contrast surround condition. A paired *t*-test confirmed that these mean PCR differences were highly significant [*t*(15) = 4.48, *p* < 0.001].

The surround contrast (high/low) by center contrast (grc/blc) ANOVA for the PCR of more dots (**Figure 4B**) revealed a significant main effect for surround contrast [*F*(1,15) = 11.81, *p* = 0.004, partial η^2^ = 0.44] but not center contrast (*F* < 1), where visual estimation of more dots was impaired by the high contrast but not low contrast surround. This surround contrast by center contrast interaction was significant [*F*(1,15) = 10.69, *p* = 0.005, partial η^2^ = 0.41], indicating that the mean PCR for estimation of more dots under zero luminance center and high contrast surround conditions for more dots, was significantly lower than that of the PCR with a gray center and high contrast surround [*t*(15) = 2.78, *p* = 0.014]. It is also worth noting that the mean PCR for estimation of more dots under gray center and low contrast surround condition was significantly lower than the PCR with black center and low contrast surround [*t*(15) = 2.40, *p* = 0.03].

The effects observed in **Figures 4A,B** suggest that the contrast of the CSR has an influential role in the perceived numerosity of dots, where it was markedly difficult for observers to discriminate less dots under high contrast surround conditions when the CSR was gray, and conversely, the ability to discriminate more dots under high contrast surround conditions was difficult for observers when the CSR was black.

### Effect of Background Contrast and Dot Set Size

**FIGURE 5:**
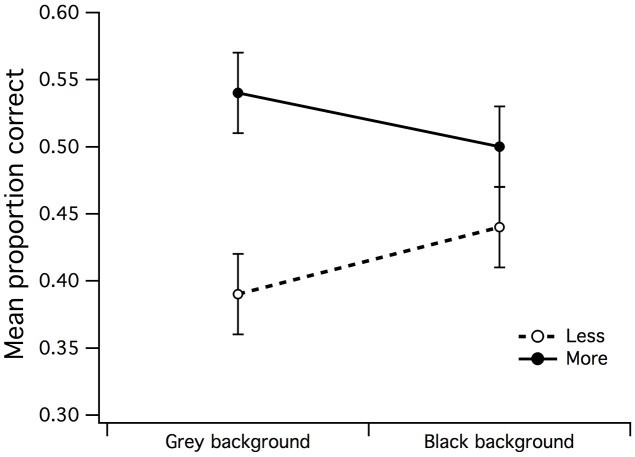
**Main effects of background luminance by dot set**. A significant main effect emerged for dot set but not for background luminance, indicative that the uniform/gray luminance background made it slightly easier for observers to accurately estimate that there were more dots in CSR3. The vertical bars at the end of each line graph represent the SE for mean PCR of fewer/more dots with gray background, and mean PCR of fewer/more dots with black background.

A 2 (gray/black background) by 2 (less/more dots) within-subjects ANOVA was run to examine the differences in mean PCR when a surround did not envelop the CSR. There was a significant main effect for set size of dots [*F*(1,15) = 6.21, *p* = 0.025, partial η^2^ = 0.29] but not for background region (*F* < 1), where it was easier for observers to accurately discriminate more dots under gray center/gray background stimulus configuration (see **Figure 5**). The background by dot set size interaction was also not significant (*F* < 1).

### Effects of Surround/Black Background and Dot Set Size

**FIGURE 6:**
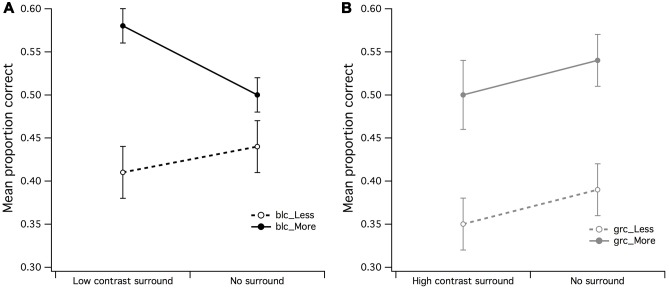
**Main effects of surround/no surround by dot set. (A)** It was apparent by the higher mean proportion of correct responses (PCR) for estimation of more dots within the low contrast surround, that observers were able to accurately estimate when there were more dots in CSR3, **(B)** a significant main effect for dot set but not surround contrast for gray central stimulus region. The vertical bars at the end of each line graph represent the SE for **(A)** mean PCR for low contrast surround and no surround (less/more dots), and **(B)** mean PCR for high contrast surround and no surround (less/more dots).

In order to examine in more detail whether the effects of surround contrast on visual estimation were distinguishable from those of background luminance (no surround), a 2 (high contrast surround/zero luminance background) by 2 (less dots/more dots) ANOVA for black center revealed that there were no significant main effects for neither surround (*F* < 1) or set of dots (*F* < 1). If no significant differences exist between the mean PCR for high contrast surround and black background it suggests that indeed, the black background luminance had the same effect as the high contrast surround. In order to confirm the inhibitory effects of the black background upon estimation judgments of dots within a black CSR, a surround (low contrast surround/black background) by dot set (less dots/more dots) within-subjects ANOVA was run. There was a significant main effect for dot set [*F*(1,15) = 7.07, *p* = 0.018, partial η^2^ = 0.32] but not for surround (*F* < 1), meaning that estimation judgments of more dots were easier for observers under black center/low contrast surround conditions (see **Figure 6A**). The surround by dot set size interaction was not significant (*F* < 1).

### Effects of Surround/Gray Background and Dot Set Size

The final set of within-subjects ANOVAs tested differences in mean PCR for surround (high and low contrast) and no surround of the gray center/gray background stimulus configuration. A 2 (low contrast surround/uniform luminance background) by 2 (less dots/more dots) ANOVA for gray center revealed that there were no significant main effects for surround (*F* < 1) or set of dots (*F* < 1). These findings suggest that the low contrast surround that enveloped the CSR3 with a gray center had the same effect as the gray background with no surround.

A 2 (high contrast surround/no surround) by 2 (less dots/more dots) ANOVA for gray center revealed a highly significant main effect for dot set [*F*(1,15) = 9.25, *p* = 0.008, partial η^2^ = 0.38] but not for surround (*F* < 1), meaning that irrespective of surround conditions (high contrast surround/gray background), it was once again easier for observers to make discrimination judgments of more dots (see **Figure 6B**). In particular, it was apparent that the mean PCR of fewer dots for high contrast surround and no surround was markedly lower than the PCR for more dots.

## Discussion

Here we investigated the effect of peripheral visual stimulation varying in sensory load on the ability to make numerosity estimation judgments of centrally presented elements. The form of peripheral visual stimulation implemented here—surround-masking, has been consistently demonstrated in earlier psychophysical literature to impair the contrast discrimination of centrally embedded texture regions, making them appear dimmer to the observer than veridically so (Chubb et al., 1989; Xing and Heeger, 2000, 2001; Dakin et al., 2005). The perceptual inefficiency induced by surround-masking has been postulated to arise from contrast gain saturation, that is, a swamping of available sensory filtering or RF inhibitory resources that serve to attenuate contextually uninformative or noisy input (Carandini, 2004; Dakin et al., 2005; Webb et al., 2005). In view of this, we expected that high contrast gain of the surrounding stimulus, would, through RF suppressive mechanisms, impair visual estimation ability of neurotypical observers. As expected, the mean PCR across observers was significantly lower during visual estimation judgments of centrally presented elements embedded in a high contrast surround annulus, compared to low contrast and no surround configurations.

To the best of our knowledge, investigation into the relationship between RF inhibitory resource limits and the perception of non-symbolic number representation is novel. However, this investigation was initiated not only from the earlier psychophysical literature on surround-masking, but also from our preliminary electrophysiological and psychophysical evidence recorded in a population of young adults with self-reported difficulty in mathematics, that displayed greater contrast saturation levels of their VEPs during high contrast gain, compared to an age matched control group who reported no difficulty with mathematics (Jastrzebski et al., 2015). Also, the math-impaired individuals in that study displayed significantly delayed visual inspection times (stimulus duration thresholds) in the accurate change detection of high Michelson contrast multi-digit numbers, compared with the control (math unimpaired) group. In combination, the lack of VEP response saturation at high contrast, and the impairment in change detection of numerals under high but not low contrast conditions, are indicative of a relationship between poorer RF inhibitory mechanisms or reduced sensory gating resources, and mathematical impairment.

It is worth pondering the relation between individual differences in visual estimation ability and the functional quality of RF suppressive mechanisms, and their influence upon the cognitive development of higher-order mathematical computations such as arithmetical or multiplicative operations. In relation to the observations of Halberda et al. (2008) that visual estimation proficiency of pre-school aged children predicts competency with high-order mathematics later in development, it is possible that the high contrast surround induced one of the behavioral characteristics of DD by limiting the available RF inhibitory (noise exclusion) resources of neurotypical observers. Thus it is conceivable that DD may receive a contribution from abnormal neurodevelopment of noise exclusion mechanisms (Johnson, 2011).

It was found that center/surround contrast configuration influenced visual estimation of more and less dots differently—with more dots being easier to discriminate within a black center/low contrast surround, and less dots being more difficult to discriminate within a gray center/high contrast surround—suggestive of a center/surround contrast interaction with influence upon the perceived numerosity of elements within the stimulus display. For the estimation of more dots during black center/low contrast surround condition (see **Figure 3A**), it is conceivable that this center/surround configuration created the illusion that the dots in CSR3 were more “numerous” than the dots in CSR1, where an earlier psychophysical investigation into visual estimation revealed that the perceived numerosity was affected by luminance of dots (Ross and Burr, 2010). Such effects of luminance were found to increase the perceived numerosity of dots with decreasing luminance. Alternatively, it was likely that the effects of center and surround contrast had increased the SNR of afferent input, thereby lowering the perceptual ambiguity in the difference between CSR1 and CSR3. The opposite center-surround effects on visual estimation observed in **Figures 3B** and **4A** indicate either that the high contrast surround also created the illusion of more dots than veridically, or that it lowered the SNR of afferent input by swamping the available inhibitory RF resources, hence creating noisiness or scalar variability in the discriminability between CSR1 and CSR3 (Gallistel and Gelman, 2000).

## Conclusion

Here we temporarily induced an impaired ability to make visual estimation judgments in neurotypical observers through high contrast surround-masking. The study has yielded novel evidence, establishing an initial link between the functional quality of inhibitory mechanisms, likely to reside in LGN/V1, and visual estimation ability. Our findings further suggest that the weak visual estimation skills observed in DD children (Piazza et al., 2010) is unlikely to derive from an innate defect in the cognitive representation of “more or less” in itself, but rather, a visuo-perceptual disorder commonly observed in those with developmental disorders such as WS (Braddick and Atkinson, 2011), ASD (Sutherland and Crewther, 2010), and neuropsychiatric disorders such as schizophrenia (Uhlhaas and Singer, 2010).

## Author Contributions

NJ wrote the manuscript, collected the data, developed the experiments, and analyzed the data. LH assisted with development of the psychophysical experiments, and proof read earlier drafts of the manuscript. SC and DC proof read each draft of the manuscript and assisted with figures.

## Conflict of Interest Statement

The authors declare that the research was conducted in the absence of any commercial or financial relationships that could be construed as a potential conflict of interest.
